# Deep-Learning ^18^F-FDG Uptake Classification Enables Total Metabolic Tumor Volume Estimation in Diffuse Large B-Cell Lymphoma

**DOI:** 10.2967/jnumed.120.242412

**Published:** 2021-01

**Authors:** Nicolò Capobianco, Michel Meignan, Anne-Ségolène Cottereau, Laetitia Vercellino, Ludovic Sibille, Bruce Spottiswoode, Sven Zuehlsdorff, Olivier Casasnovas, Catherine Thieblemont, Irène Buvat

**Affiliations:** 1 Siemens Healthcare GmbH, Erlangen, Germany; 2 Technical University of Munich, Munich, Germany; 3 Lysa Imaging, Henri Mondor University Hospitals, APHP, University Paris East, Créteil, France; 4 Department of Nuclear Medicine, Cochin Hospital, AP-HP, Paris, France; 5 Department of Nuclear Medicine, Saint-Louis Hospital, AP-HP, Paris, France; 6 Siemens Medical Solutions USA, Inc., Knoxville, Tennessee; 7 Department of Hematology, University Hospital of Dijon, Dijon, France; 8 Department of Hematology, Saint Louis Hospital, APHP, Paris, France; and; 9 Laboratoire d’Imagerie Translationnelle en Oncologie, INSERM, Institut Curie, Université Paris–Saclay, Orsay, France

**Keywords:** metabolic tumor volume, lymphoma, deep learning, FDG, PET/CT

## Abstract

Total metabolic tumor volume (TMTV), calculated from ^18^F-FDG PET/CT baseline studies, is a prognostic factor in diffuse large B-cell lymphoma (DLBCL) whose measurement requires the segmentation of all malignant foci throughout the body. No consensus currently exists regarding the most accurate approach for such segmentation. Further, all methods still require extensive manual input from an experienced reader. We examined whether an artificial intelligence–based method could estimate TMTV with a comparable prognostic value to TMTV measured by experts. **Methods:** Baseline ^18^F-FDG PET/CT scans of 301 DLBCL patients from the REMARC trial (NCT01122472) were retrospectively analyzed using a prototype software (PET Assisted Reporting System [PARS]). An automated whole-body high-uptake segmentation algorithm identified all 3-dimensional regions of interest (ROIs) with increased tracer uptake. The resulting ROIs were processed using a convolutional neural network trained on an independent cohort and classified as nonsuspicious or suspicious uptake. The PARS-based TMTV (TMTV_PARS_) was estimated as the sum of the volumes of ROIs classified as suspicious uptake. The reference TMTV (TMTV_REF_) was measured by 2 experienced readers using independent semiautomatic software. The TMTV_PARS_ was compared with the TMTV_REF_ in terms of prognostic value for progression-free survival (PFS) and overall survival (OS). **Results:** TMTV_PARS_ was significantly correlated with the TMTV_REF_ (ρ = 0.76; *P* < 0.001). Using PARS, an average of 24 regions per subject with increased tracer uptake was identified, and an average of 20 regions per subject was correctly identified as nonsuspicious or suspicious, yielding 85% classification accuracy, 80% sensitivity, and 88% specificity, compared with the TMTV_REF_ region. Both TMTV results were predictive of PFS (hazard ratio, 2.3 and 2.6 for TMTV_PARS_ and TMTV_REF_, respectively; *P* < 0.001) and OS (hazard ratio, 2.8 and 3.7 for TMTV_PARS_ and TMTV_REF_, respectively; *P* < 0.001). **Conclusion:** TMTV_PARS_ was consistent with that obtained by experts and displayed a significant prognostic value for PFS and OS in DLBCL patients. Classification of high-uptake regions using deep learning for rapidly discarding physiologic uptake may considerably simplify TMTV estimation, reduce observer variability, and facilitate the use of TMTV as a predictive factor in DLBCL patients.

Total metabolic tumor volume (TMTV) derived from ^18^F-FDG PET/CT baseline studies is a promising prognostic factor in diffuse large B-cell lymphoma (DLBCL) (1,2) and other types of lymphoma (3–5). DLBCL is the most frequent non-Hodgkin lymphoma, being present in about 30%–40% of non-Hodgkin lymphoma cases worldwide. Although the prognosis of DLBCL can be improved with immunochemotherapy, more than 30% of patients are refractory or relapse after first-line treatment, with a poor outcome (6,7). Therefore, there is a need to identify high-risk patients who could benefit from intensive or novel therapies early. Unfortunately, the role of current prognostic factors such as the International Prognostic Index (8), Revised International Prognostic Index (9), and National Comprehensive Cancer Network International Prognostic Index (10), based on tumor burden surrogates is limited. Thus, baseline TMTV, which estimates the total metabolic tumor burden at diagnosis, has been proposed as an alternative prognostic tool for early risk stratification.

To date, TMTV is not yet routinely used in clinical lymphoma patient management, in part because of a lack of consensus throughout the literature. Several methods have been proposed to calculate TMTV (11–13), and the cutoffs reported to detect high-risk patients differed among methods and studies. However, recent studies have suggested that, despite these differences, most methods yielded similar accuracy in predicting patient prognosis when applied in similar patient groups (11,12), emphasizing the strong prognostic power of baseline TMTV.

Regardless of the criteria used for delineating tumor regions, all methods for deriving TMTV require extensive and time-consuming manual input from an experienced reader. The reader either manually segments the tumor regions or, more commonly, uses an automated method to detect all regions with increased uptake and then manually eliminates the regions of physiologic uptake and adds in undetected tumor regions (13). Recently, a machine-learning algorithm using a convolutional neural network (CNN) was trained to differentiate physiologic from nonphysiologic uptake regions in whole-body ^18^F-FDG PET scans acquired from an unselected population of more than 600 patients, including half who were lymphoma patients with different subtypes of diseases (14,15). This CNN achieved a high degree of accuracy in characterizing increased tracer uptake in the whole body as physiologic or nonphysiologic. Such automated identification of nonphysiologic regions would facilitate TMTV measurement and clinical adoption. This study therefore sought to assess the ability of this CNN to identify regions from which TMTV could be automatically calculated and to evaluate the ability of the resulting TMTV in predicting patient outcome among a large group of DLBCL patients included in an international phase III trial wherein TMTV has already been demonstrated to be a strong predictor of 4-y progression-free survival (PFS) and overall survival (OS). To evaluate the CNN performance, regions with elevated tracer uptake automatically identified as physiologic or suspicious were compared with regions attributed to suspicious uptake by an expert reader using a semiautomatic method.

## MATERIALS AND METHODS

### Patients

Patients from an ancillary study (16,17) of the REMARC trial (NCT01122472) were retrospectively analyzed. This trial is a phase III study that was designed to assess the efficacy of lenalidomide versus placebo in responding elderly DLBCL patients (60–80 y old) treated with the standard first-line rituximab, cyclophosphamide, doxorubicin hydrochloride (hydroxydaunorubicin), vincristine sulfate, and prednisone (R-CHOP) therapy approach (18). The institutional review board approval and the informed consent of the REMARC trial included all the ancillary studies. The ancillary study was conducted by involving 301 patients who underwent baseline PET/CT before R-CHOP and showed that TMTV was a strong prognosticator of outcome in patients responding to first-line chemotherapy combined with monoclonal antibody treatment.

### Image Acquisition and Analysis

All baseline ^18^F-FDG PET/CT images from the ancillary study were collected in an anonymized DICOM format. Patients whose PET or CT DICOM series had incomplete axial slices or irregular slice intervals were excluded. PET images were expressed in SUV units, accounting for injected dose and patient body weight.

PET/CT images were analyzed using an investigational software prototype (PET Assisted Reporting System [PARS]; Siemens Medical Solutions USA, Inc.) that uses artificial intelligence. The prototype first automatically located a cylindric reference region at the center of the proximal descending aorta by applying a landmarking algorithm to the CT image (19). This region was used to determine the mean blood pool SUV and mean blood pool SUV standard deviation (SD), following PERCIST recommendations (20). The 3-dimensional regions of the PET image with increased tracer uptake were identified for each subject using an automated whole-body high-uptake segmentation algorithm (multi-foci segmentation, MFS) (21). In line with the PERCIST recommendations, only the regions with SUV_peak_ greater than twice the mean blood pool SUV plus twice the mean blood pool SUV SD were included. Those regions were then further segmented according to 42% of the SUV_max_ threshold, and the ones with volumes below 2 cm^3^ were discarded. The resulting regions of interest (ROIs), called ROI_PARS_, were then automatically processed by a CNN. Details of the training and validation of this CNN were previously reported (15). The input of the CNN was the PET/CT data together with the set of ROI_PARS_ sites. For each ROI_PARS_, the output of the CNN was the anatomic localization among a set of possible anatomic sites relevant for staging and whether the ROI_PARS_ uptake was physiologic (e.g., due to unspecific bowel uptake, muscle activation, inflammation, infection, or bone degeneration) or suspicious (i.e., due to lymphoma). The volumes of all ROI_PARS_ sites classified as suspicious uptake were then summed to obtain the TMTV_PARS_.

The CNN was also used in combination with 2 other settings of the initial high-uptake ROI segmentation: the first used an initial threshold of 2.5 SUV instead of the blood-pool–based threshold, followed by thresholding with 41% of SUV_max_; the second also included ROIs with a volume between 0.1 and 2 cm^3^.

The TMTV obtained by 2 experienced nuclear medicine physicians in the context of a previous study (16,17) was used as a reference (TMTV_REF_). The TMTV_REF_ was obtained using the semiautomatic version of the Beth Israel Fiji (ImageJ) software plugin (22), which was previously used to demonstrate the prognostic value of TMTV in various lymphoma subtypes (5,23). To calculate TMTV_REF_, the physician combined automated and manual steps as follows. First, volumes of interest with high uptake in the PET images were segmented using an automated method, which applied in sequence an algorithm based on component trees and shape priors (24), a region growing, and a final region delineation using 41% of the region SUV_max_ threshold (25). Second, the resulting ROIs were manually reviewed by the reader, who selected only the regions corresponding to lymphoma (ROI_REF_), adding an ROI_REF_ wherever a lymphoma lesion had been missed by the algorithm by drawing a prism around that lesion and applying a 41% SUV_max_ threshold. The volumes of all lymphoma ROI_REF_ sites were summed to obtain the reference TMTV (TMTV_REF_).

### Statistical Analysis

To evaluate the performance of the CNN classification, for each patient, each ROI_PARS_, having been labeled as presenting suspicious or physiologic uptake by the CNN, was compared with all the ROI_REF_ sites of that patient taken together. The ROI_PARS_ was considered to match the ROI_REF_ if at least 50% of its volume overlapped with one or several ROI_REF_ sites. ROI_PARS_ sites classified as suspicious and matching one or several ROI_REF_ sites were considered true-positives, ROI_PARS_ sites classified as physiologic and matching one or several ROI_REF_ sites were considered false-negatives, ROI_PARS_ sites classified as physiologic and not matching any ROI_REF_ sites were considered true-negatives, and ROI_PARS_ sites classified as suspicious and not matching any ROI_REF_ sites were considered false-positives. The sensitivity, specificity, and accuracy of the uptake classification were calculated. The performance of the CNN classification was also assessed in case a minimum overlap of 25% and 75% was required to consider an ROI_PARS_ as matching the ROI_REF_.

To evaluate differences between TMTV_PARS_ and TMTV_REF_, Bland–Altman analysis was performed. Since the Shapiro–Wilk test revealed a significant nonnormal distribution of the differences between TMTV_PARS_ and TMTV_REF_ (*P* < 0.001), the median bias and limits of agreement at the 2.5 and 97.5 percentiles were reported in the Bland–Altman plot. To assess the correlation between ranked TMTV values, the Spearman rank correlation coefficient was used. For each patient, the agreement between the patient set of ROI_PARS_ sites classified as suspicious and the patient set of ROI_REF_ sites was characterized using the Dice score, precision (the fraction of voxels in the set of ROI_PARS_ sites classified as suspicious that were also present in the set of ROI_REF_ sites), and recall (the fraction of voxels in the set of ROI_REF_ sites that were also present in the set of ROI_PARS_ sites classified as suspicious).

Survival analysis was performed for both TMTV_PARS_ and TMTV_REF_ with respect to PFS and OS. Receiver-operating-characteristic curves were used to determine TMTV cutoffs to predict the occurrence of events within 4 y for both PFS and OS, by maximizing the Youden index (sensitivity + specificity − 1). Survival functions were computed by Kaplan–Meier analyses and used to estimate survival time statistics (such as 4-y PFS rate and 4-y OS rate) for low- and high-TMTV groups. A log-rank test was used to assess whether differences between Kaplan–Meier survival curves were significant. Univariate Cox regression was used to calculate hazard ratios between survival groups. Statistical significance was set at a *P* value of less than 0.05. Statistical analysis was performed using R, version 3.6.1, with survivalROC, version 1.0.3, and pROC, version 1.15.3 (26).

## RESULTS

In total, 280 patients from 124 centers were included in the analysis. Patient characteristics are reported in Table 1. All received first-line treatment with R-CHOP and were responders at the time of inclusion in the trial, 142 received a lenalidomide regimen afterward as maintenance, and 138 received placebo. After a median follow-up of 5 y, 86 patients presented with a PFS event and 51 patients had an OS event; the 4-y survival rates were 69% for PFS and 83% for OS. The 4-y survival rates were comparable to those of the entire trial.

**TABLE 1:** Patient Characteristics

Patient characteristics	Data
Sex	
Female	119 (42.5)
Male	161 (57.5)
Age (y)	
Median	68
Range	58–80
Ann Arbor stage	
I	1 (0.4)
II	25 (8.9)
III	57 (20.4)
IV	197 (70.4)
Performance status*	
0	113 (40.4)
1	119 (42.5)
2	39 (13.9)
3	2 (0.7)
4	2 (0.7)
Missing	5 (1.8)
International Prognostic Index	
1	6 (2.1)
2	73 (26.1)
3	97 (34.6)
4	81 (28.9)
5	19 (6.8)
Missing	4 (1.4)
Elevated lactate dehydrogenase^†^	
No	111 (39.6)
Yes	165 (58.9)
Missing	4 (1.4)

* Eastern Cooperative Oncology Group.

† Greater than upper limit of normal set specifically for each laboratory.

Data are *n* followed by percentage in parentheses, except for age. Total *n* is 280.

PET/CT images were acquired using different scanner models from different vendors as summarized in Supplemental Table 1 (supplemental materials are available at http://jnm.snmjournals.org). The delay between injection and acquisition time was 71.7 ± 14.1 min (mean ± SD). The SUV_mean_ in the proximal descending aorta cylindric region was 1.6 ± 0.5 (mean ± SD across subjects), resulting in an SUV_peak_ threshold of 3.6 ± 1.2 for detecting ROIs with increased tracer uptake.

The results below are described for the PERCIST-based setting of the initial high-uptake ROI segmentation, whereas changes observed with other settings are reported in Supplemental Tables 2–4.

### Uptake Classification

In total, 6,737 ROI_PARS_ sites exhibiting increased uptake were obtained from the 280 subjects. There were 7,996 ROI_REF_ sites in the 280 subjects. Descriptive statistics for the number of ROI_PARS_ and ROI_REF_ sites per subject are summarized in Supplemental Table 5. Among the 6,737 ROI_PARS_ sites with increased uptake, 2,831 (42%) were classified as having suspicious uptake by the CNN.

When compared with the ROI_REF_ sites obtained by the experienced reader, the identification of the ROI_PARS_ sites with suspicious uptake by the CNN yielded 3,317 true-negatives, 2,399 true-positives, 589 false-negatives, and 432 false-positives. Corresponding sensitivity was 80%, specificity was 88%, and accuracy was 85%.

**FIGURE 1.**
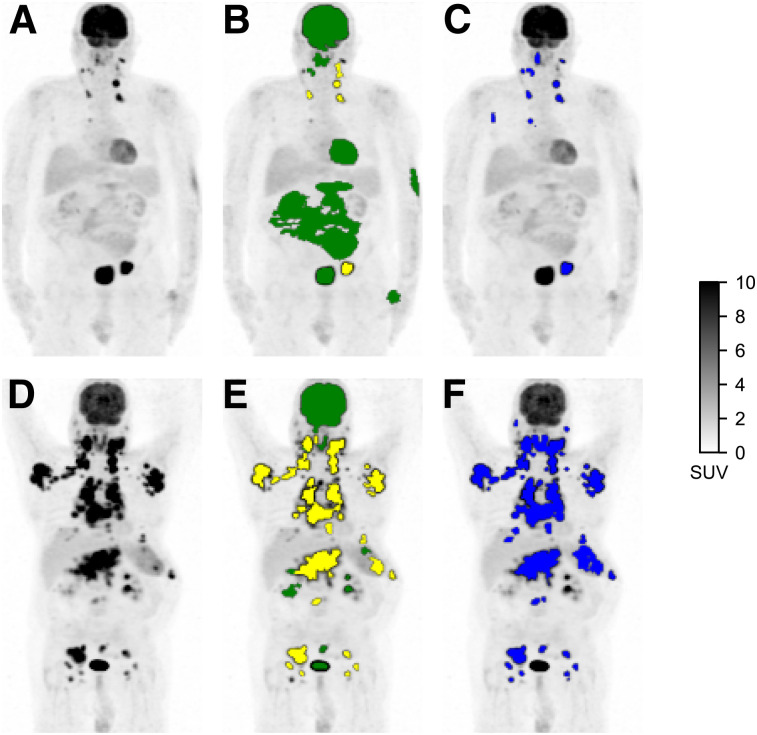
Detection of regions of high ^18^F-FDG uptake and classification as physiologic or suspicious. (A and D) Maximum-intensity-projection PET images of subjects with low TMTV (A) and high TMTV (D). (B and E) ROI_PARS_ obtained automatically using PARS software prototype. ROI_PARS_ sites detected by MFS algorithm are overlaid onto PET maximum-intensity projection. ROI_PARS_ sites classified by deep-learning algorithm as physiologic are shown in green, and ROI_PARS_ sites classified as suspicious are shown in yellow. (C and F) ROI_REF_ obtained by an experienced nuclear medicine physician using semiautomatic software.

Additionally, the mean per-subject ROI_PARS_ classification accuracy was 87% (median, 89%; interquartile range [IQR], 81%–96%). There were an average of 20 correctly classified ROI_PARS_ sites per subject (median, 17 ROI_PARS_ sites; IQR, 11–27 ROI_PARS_ sites) and an average of 4 incorrectly classified ROI_PARS_ sites per subject (median, 2 ROI_PARS_ sites; IQR, 1–5 ROI_PARS_ sites), which were regions classified as suspicious by the CNN that did not overlap with the set of ROI_REF_ sites or regions classified as physiologic by the CNN but overlapped with the set of ROI_REF_ sites. Two examples of uptake classification of ROI_PARS_ sites with corresponding ROI_REF_ are shown in Figure 1. Results with a minimum overlap of 25% and 75% required to consider a ROI_PARS_ as matching the ROI_REF_ are reported in Supplemental Table 6.

### TMTV

After discarding the ROI_PARS_ sites classified as physiologic uptake by the CNN, a median TMTV_PARS_ of 110 cm^3^ was obtained (IQR, 33–281 cm^3^). The median TMTV_REF_ was 240 cm^3^ (IQR, 80–529 cm^3^) (Table 2).

**TABLE 2:** Statistics for TMTV Using PARS and Reference Method

TMTV Estimation	Mean	SD	Minimum	Q1 (25%)	Median	Q3 (75%)	Maximum
TMTV_PARS_ (cm^3^)	235.2	347.6	0.0	32.9	110.2	280.8	2471.9
TMTV_REF_ (cm^3^)	433.7	571.3	2.27	80.0	240.0	529.3	3832.7

**FIGURE 2.**
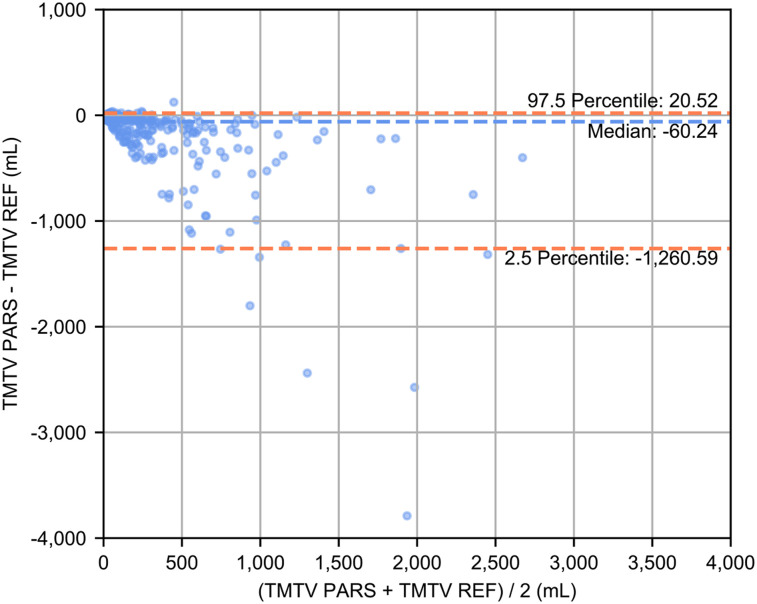
Bland–Altman plot comparing TMTV obtained using PARS and TMTV_REF_ obtained by nuclear medicine physician using semiautomatic software.

There was a significant correlation between ranked TMTV estimates (ρ = 0.76; *P* < 0.001). The median Dice score across all patients between the patient set of ROI_PARS_ sites labeled as suspicious and the patient set of ROI_REF_ sites was 0.73 (IQR, 0.33–0.86), the median recall of the patient set of ROI_PARS_ sites labeled as suspicious with respect to the patient set of ROI_REF_ sites was 0.62 (IQR, 0.20–0.81), and the median precision was 0.96 (IQR, 0.86–0.99). The Bland–Altman plot comparing TMTV_PARS_ and TMTV_REF_ (Fig. 2) showed wide limits of agreement.

### Survival Analysis

**FIGURE 3.**
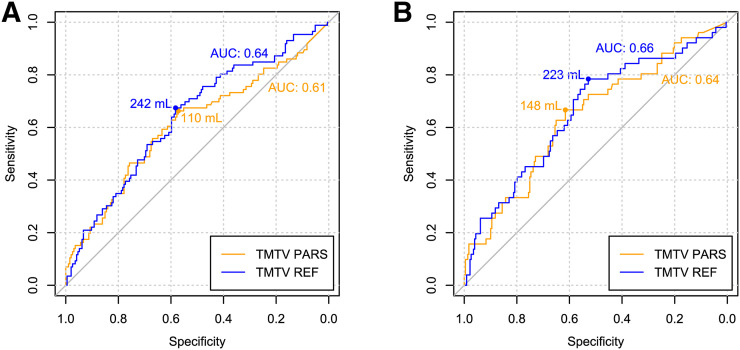
Receiver-operating-characteristic curves for TMTV_PARS_ and TMTV_REF_ for 4-y PFS (A) and 4-y OS (B). Areas under receiver-operating-characteristic curves (AUC) and optimal TMTV cutoffs are reported.

**FIGURE 4.**
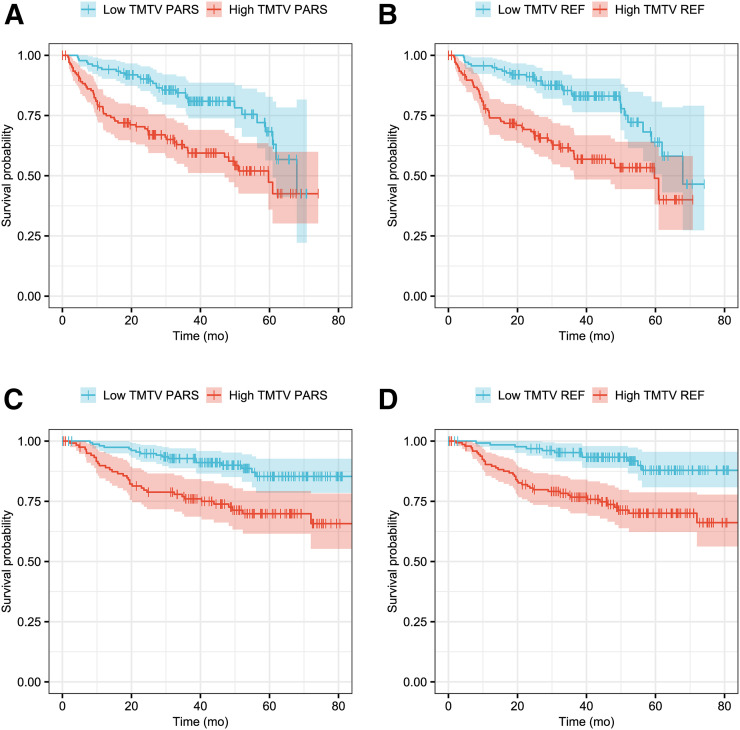
Kaplan–Meier survival curves for PFS (A and B) and OS (C and D).

The area under the receiver-operating-characteristic curve for predicting the 4-y PFS was 0.63 for TMTV_PARS_ and 0.69 for TMTV_REF_ (Fig. 3). The optimal cutoffs for predicting the 4-y PFS were 171 cm^3^ for TMTV_PARS_ and 242 cm^3^ for TMTV_REF_. Kaplan–Meier survival curves are shown in Figure 4. The 4-y PFS rates were 79% and 54% for the low- and high-TMTV_PARS_ groups and 83% and 55% for the low- and high-TMTV_REF_ groups, respectively. The log-rank test indicated a significantly longer PFS time in the low-TMTV patient group for both TMTV estimation methods (*P* < 0.001 for TMTV_PARS_ and TMTV_REF_). Cox regression for PFS resulted in hazard ratios (high-TMTV group vs. low-TMTV group) of 2.3 (95% confidence interval, 1.5–3.6; *P* < 0.001 for Wald test) for TMTV_PARS_ and 2.6 (95% confidence interval, 1.6–4.1; *P* < 0.001) for TMTV_REF_. The survival results are summarized in Table 3.

**TABLE 3:** TMTV AUC, Hazard Ratio, and 4-Year Survival Analyses for PFS and OS

TMTV estimation	AUC	Cutoff (cm^3^)	Hazard ratio	High TMTV 4-y survival	Low TMTV 4-y survival	*P*
PFS						
TMTV_PARS_	0.63	171	2.3 (1.5–3.6)	54%	79%	0.00009
TMTV_REF_	0.69	242	2.6 (1.6–4.1)	55%	83%	0.00004
OS						
TMTV_PARS_	0.65	148	2.8 (1.6–5.1)	74%	90%	0.00044
TMTV_REF_	0.68	223	3.7 (1.9–7.2)	74%	93%	0.00012

AUC = area under receiver-operating-characteristic curve.

Data in parentheses are 95% confidence intervals.

For the 4-y OS, the area under the receiver-operating-characteristic curve was 0.65 for TMTV_PARS_ and 0.68 for TMTV_REF_. The optimal TMTV cutoffs for predicting the 4-y OS were 148 cm^3^ for TMTV_PARS_ and 223 cm^3^ for TMTV_REF_. The 4-y OS rates were 90% and 74% for the low- and high-TMTV_PARS_ groups and 93% and 74% for the low- and high-TMTV_REF_ groups, respectively. The log-rank test revealed a significantly higher OS time in the low-TMTV patient group for both TMTV estimation methods (*P* < 0.001 for TMTV_PARS_ and TMTV_REF_). Cox regression for OS resulted in hazard ratios (high-TMTV group vs. low-TMTV group) of 2.8 (95% confidence interval, 1.6–5.1; *P* < 0.001) for TMTV_PARS_ and 3.7 (95% confidence interval, 1.9–7.2; *P* < 0.001) for TMTV_REF_.

The sensitivity, specificity, negative predictive value, positive predictive value, and accuracy for predicting the occurrence of survival events within 4 y, determined at the optimal TMTV cutoff for each method, are reported in Supplemental Table 7 and were similar for both PFS and OS.

## DISCUSSION

Our main result was that a fully automated method combining a region delineation method based on PERCIST recommendations and a CNN-based algorithm to distinguish between regions with elevated physiologic uptake and nonphysiologic regions was able to generate, in a uniform population of DLBCL patients, TMTV values predictive of 4-y PFS and OS with an accuracy comparable to that obtained when TMTV is calculated by manual selection of the tumor regions by medical experts. Although the CNN-based algorithm was trained using images obtained on only 2 scanner models from the same vendor, the algorithm was highly accurate in classifying increased uptake in patients from an international trial involving 124 centers that obtained images on different scanner models from different vendors and with variable reconstruction settings. This accuracy underlines the robustness of the CNN despite different image quality. Moreover, this algorithm was not originally trained for TMTV computation and outcome prediction and was developed with data from patients with different lymphoma subtypes and lung cancer who underwent PET at baseline and for response assessment. However, we showed that the algorithm was successful in a group of patients with a homogeneous lymphoma subtype scanned at baseline, enabling the identification of a TMTV cutoff separating high-risk from low-risk patients and predicting prognosis with accuracy comparable to that of the reference method. No subject was excluded because of failure of the initial high-uptake ROI segmentation, which identified at least one high-uptake region in all subjects. Furthermore, comparable results were obtained when different settings of the initial high-uptake ROI segmentation were applied using a lower threshold (2.5 SUV) than the PERCIST-recommended blood-pool–based threshold (Supplemental Tables 2 and 3), suggesting the robustness of the algorithm to the initial segmentation results. Additionally, the accuracy of the high-uptake ROI classification was not substantially impacted when a different level of overlap was required to consider an ROI as matching the TMTV_REF_ and when ROIs with volumes of less than 2 cm^3^ were included in the analysis (Supplemental Tables 4 and 6).

The median TMTV_PARS_ and the resulting cutoff were lower than those observed for TMTV_REF_. This finding could be due to multiple factors, including the higher initial SUV threshold used for TMTV_PARS_ relative to the one used for TMTV_REF_, the manual addition of suspicious regions with low uptake in TMTV_REF_, regions being classified as physiologic in TMTV_PARS_ but considered suspicious in TMTV_REF_, and differences in the contouring of suspicious regions between TMTV_PARS_ and TMTV_REF_. However, the ability of the TMTV_PARS_ estimates to be predictive of PFS and OS despite involving a TMTV range different from that of TMTV_REF_ is consistent with what has already been reported (11,12) when comparing different TMTV estimation methods. This result confirms both the validity of the CNN method and the value of TMTV as a prognostic indicator.

Our study had limitations. Since there is currently no gold standard method for TMTV calculation from ^18^F-FDG PET/CT images (27), the reported figures of merit supporting the uptake classification performance and accuracy of the TMTV segmentation are limited to the comparison with the reference method considered in the study. Moreover, a uniform cohort of lymphoma patients was evaluated in the current study, and results may differ for different lymphoma subtypes or different cancer types.

In the present work, we evaluated a fully automated application of PARS. However, PARS was initially intended to be used in a supervised manner, allowing the reader to correct for potentially misclassified regions when appropriate. In particular, pitfalls in PET/CT image quality, such as misalignment due to motion or image artifacts, may influence the classification output of the CNN algorithm, and the results should be validated by an expert. This is especially true when the labeling results are used to derive a prognostic index such as TMTV that can be used to stratify the risk and guide personalized therapy. Nevertheless, this approach could be used by expert readers to efficiently estimate TMTV, as the deep-learning–based method is able to automatically identify several relevant suspicious uptake sites and automatically discard physiologic uptake sites, with the expert only having to correct the potential improper classification of a limited number of regions per subject, requiring limited user interaction and potentially improving interreader variability. This approach may introduce bias in the TMTV estimation process by relying on pregenerated results. However, this risk should be marginal, especially when a careful revision of the results is performed by an experienced reader.

To our knowledge, this was the first study showing that an artificial intelligence method can generate a TMTV value prognostic of outcome in a large series of patients with DLBCL, with results comparable to other currently used methodologies. Other machine-learning–based approaches for TMTV estimation in lymphoma patients, including some involving CNN, are being developed and evaluated (28). The automated method for TMTV segmentation assessed in the present study combined a region-delineation method based on PERCIST recommendations and a deep-learning–based classification scheme for rapidly discarding physiologic uptake. Further efforts toward developing a stricter definition of TMTV, standardizing volume-segmentation methods, and establishing guidelines for the inclusion of tumor-bearing anatomic regions are ongoing, and these will constitute a prerequisite for the optimization of a complete automated method (13).

## CONCLUSION

We showed that TMTV can be estimated fully automatically using a deep-learning approach. The resulting TMTV was consistent with that obtained by independent experts and showed significant prognostic value for PFS and OS in a large cohort of DLBCL subjects.

## DISCLOSURE

This project received funding from the European Union’s Horizon 2020 research and innovation program under the Marie Skłodowska–Curie grant agreement (grant 764458). Nicolò Capobianco is a full-time employee of Siemens Healthcare GmbH. Ludovic Sibille, Bruce Spottiswoode, and Sven Zuehlsdorff are full-time employees of Siemens Medical Solutions USA, Inc. No other potential conflict of interest relevant to this article was reported.

**KEY POINTS**

**QUESTION:** Can deep learning be used to obtain an automated estimation of TMTV in baseline ^18^F-FDG PET/CT for risk stratification in DLBCL patients?
**PERTINENT FINDINGS:** In a cohort of 280 DLBCL patients from the REMARC trial, a deep-learning algorithm could classify regions of interest with elevated uptake in ^18^F-FDG PET/CT as physiologic or suspicious in good agreement with expert human reader assessment. By aggregating the regions of interest classified as suspicious uptake by the deep-learning algorithm, the automated TMTV estimates were significant for PFS and OS prediction.
**IMPLICATIONS FOR PATIENT CARE:** Estimation of TMTV with an automated method using deep learning may contribute to reproducible and accurate identification of high-risk patients with DLBCL.

## References

[1] Sasanelli M Meignan M Haioun C. Pretherapy metabolic tumour volume is an independent predictor of outcome in patients with diffuse large B-cell lymphoma. Eur J Nucl Med Mol Imaging. 2014;41:2017–2022. 24902639 10.1007/s00259-014-2822-7

[2] Song M-K Chung J-S Shin H-J. Clinical significance of metabolic tumor volume by PET/CT in stages II and III of diffuse large B cell lymphoma without extranodal site involvement. Ann Hematol. 2012;91:697–703. 22071570 10.1007/s00277-011-1357-2 PMC3319905

[3] Kanoun S Rossi C Berriolo-Riedinger A. Baseline metabolic tumour volume is an independent prognostic factor in Hodgkin lymphoma. Eur J Nucl Med Mol Imaging. 2014;41:1735–1743. 24811577 10.1007/s00259-014-2783-x

[4] Cottereau AS Becker S Broussais F. Prognostic value of baseline total metabolic tumor volume (TMTV0) measured on FDG-PET/CT in patients with peripheral T-cell lymphoma (PTCL). Ann Oncol. 2016;27:719–724. 26787236 10.1093/annonc/mdw011

[5] Meignan M Cottereau AS Versari A. Baseline metabolic tumor volume predicts outcome in high–tumor-burden follicular lymphoma: a pooled analysis of three multicenter studies. J Clin Oncol. 2016;34:3618–3626. 27551111 10.1200/JCO.2016.66.9440

[6] Gisselbrecht C Glass B Mounier N. Salvage regimens with autologous transplantation for relapsed large B-cell lymphoma in the rituximab era. J Clin Oncol. 2010;28:4184–4190. 20660832 10.1200/JCO.2010.28.1618 PMC3664033

[7] Crump M Neelapu SS Farooq U. Outcomes in refractory diffuse large B-cell lymphoma: results from the international SCHOLAR-1 study. Blood. 2017;130:1800–1808. 28774879 10.1182/blood-2017-03-769620 PMC5649550

[8] International non-Hodgkin’s lymphoma prognostic factors project. A predictive model for aggressive non-Hodgkin’s lymphoma. N Engl J Med. 1993;329:987–994. 8141877 10.1056/NEJM199309303291402

[9] Sehn LH Berry B Chhanabhai M. The revised International Prognostic Index (R-IPI) is a better predictor of outcome than the standard IPI for patients with diffuse large B-cell lymphoma treated with R-CHOP. Blood. 2007;109:1857–1861. 17105812 10.1182/blood-2006-08-038257

[10] Zhou Z Sehn LH Rademaker AW. An enhanced International Prognostic Index (NCCN-IPI) for patients with diffuse large B-cell lymphoma treated in the rituximab era. Blood. 2014;123:837–842. 24264230 10.1182/blood-2013-09-524108 PMC5527396

[11] Cottereau A-S Hapdey S Chartier L. Baseline total metabolic tumor volume measured with fixed or different adaptive thresholding methods equally predicts outcome in peripheral T cell lymphoma. J Nucl Med. 2017;58:276–281. 27754905 10.2967/jnumed.116.180406

[12] Ilyas H Mikhaeel NG Dunn JT. Defining the optimal method for measuring baseline metabolic tumour volume in diffuse large B cell lymphoma. Eur J Nucl Med Mol Imaging. 2018;45:1142–1154. 29460024 10.1007/s00259-018-3953-z PMC5953976

[13] Barrington SF Meignan MA. Time to prepare for risk adaptation in lymphoma by standardizing measurement of metabolic tumour burden. J Nucl Med. 2019;60:1096–1102. 30954945 10.2967/jnumed.119.227249 PMC6681699

[14] Sibille L Avramovic N Spottiswoode B Schaefers M Zuehlsdorff S Declerck J. PET uptake classification in lymphoma and lung cancer using deep learning [abstract]. J Nucl Med. 2018;59(suppl 1):325.

[15] Sibille L Seifert R Avramovic N. ^18^F-FDG PET/CT uptake classification in lymphoma and lung cancer by using deep convolutional neural networks. Radiology. 2020;294:445–452. 31821122 10.1148/radiol.2019191114

[16] Cottereau A Vercellino L Casasnovas O. High total metabolic tumor volume at baseline allows to discriminate for survival patients in response after R-CHOP: an ancillary analysis of the REMARC study [abstract]. Hematol Oncol. 2019;37(suppl 2):49–50.

[17] Vercellino L Cottereau AS Casasnovas O. High total metabolic tumor volume at baseline predicts survival independent of response to therapy. Blood. 2020;135:1396–1405. 10.1182/blood.2019003526 PMC7162688 31978225

[18] Thieblemont C Tilly H Gomes da Silva M. Lenalidomide maintenance compared with placebo in responding elderly patients with diffuse large B-cell lymphoma treated with first-line rituximab plus cyclophosphamide, doxorubicin, vincristine, and prednisone. J Clin Oncol. 2017;35:2473–2481. 28426350 10.1200/JCO.2017.72.6984

[19] Tao Y Peng Z Krishnan A Zhou XS. Robust learning-based parsing and annotation of medical radiographs. IEEE Trans Med Imaging. 2011;30:338–350. 20876012 10.1109/TMI.2010.2077740

[20] Wahl RL Jacene H Kasamon Y Lodge MA. From RECIST to PERCIST: evolving considerations for PET response criteria in solid tumors. J Nucl Med. 2009;50:122S–150S. 19403881 10.2967/jnumed.108.057307 PMC2755245

[21] Brito A Santos A Mosci C. Comparison of manual versus semi-automatic quantification of skeletal tumor burden on ^18^F-fluoride PET/CT [abstract]. J Nucl Med. 2017;58(suppl 1):766.

[22] Kanoun S Tal I Berriolo-Riedinger A. Influence of software tool and methodological aspects of total metabolic tumor volume calculation on baseline [^18^F] FDG PET to predict survival in Hodgkin lymphoma. PLoS One. 2015;10:e0140830. 26473950 10.1371/journal.pone.0140830 PMC4608733

[23] Cottereau A-S Versari A Loft A. Prognostic value of baseline metabolic tumor volume in early-stage Hodgkin lymphoma in the standard arm of the H10 trial. Blood. 2018;131:1456–1463. 29437590 10.1182/blood-2017-07-795476

[24] Grossiord E Talbot H Passat N Meignan M Tervé P Najman L. Hierarchies and shape-space for PET image segmentation. In: *2015 IEEE 12th International Symposium on Biomedical Imaging (ISBI)*. Piscataway, NJ: IEEE; 2015:1118–1121.

[25] Meignan M Sasanelli M Casasnovas RO. Metabolic tumour volumes measured at staging in lymphoma: methodological evaluation on phantom experiments and patients. Eur J Nucl Med Mol Imaging. 2014;41:1113–1122. 24570094 10.1007/s00259-014-2705-y

[26] Robin X Turck N Hainard A. pROC: an open-source package for R and S+ to analyze and compare ROC curves. BMC Bioinformatics. 2011;12:77. 21414208 10.1186/1471-2105-12-77 PMC3068975

[27] Cottereau AS Buvat I Kanoun S. Is there an optimal method for measuring baseline metabolic tumor volume in diffuse large B cell lymphoma? Eur J Nucl Med Mol Imaging. 2018;45:1463–1464. 29651546 10.1007/s00259-018-4005-4

[28] Jemaa S Fredrickson J Coimbra A. A fully automated measurement of total metabolic tumor burden in diffuse large B-cell lymphoma and follicular lymphoma [abstract]. Blood. 2019;134(suppl 1):4666.

